# Mettl14 Attenuates Cardiac Ischemia/Reperfusion Injury by Regulating Wnt1/β-Catenin Signaling Pathway

**DOI:** 10.3389/fcell.2021.762853

**Published:** 2021-12-16

**Authors:** Ping Pang, Zhezhe Qu, Shuting Yu, Xiaochen Pang, Xin Li, Yuelin Gao, Kuiwu Liu, Qian Liu, Xiuzhu Wang, Yu Bian, Yingqi Liu, Yingqiong Jia, Zhiyong Sun, Hanif Khan, Zhongting Mei, Xiaoqian Bi, Changhao Wang, Xinda Yin, Zhimin Du, Weijie Du

**Affiliations:** ^1^ Department of Pharmacology (The State-Province Key Laboratories of Biomedicine Pharmaceutics of China, Key Laboratory of Cardiovascular Research, Ministry of Education), College of Pharmacy, Harbin Medical University, Harbin, China; ^2^ Institute of Clinical Pharmacy, The Second Affiliated Hospital of Harbin Medical University (The University Key Laboratory of Drug Research, Heilongjiang Province), Harbin, China; ^3^ Department of Clinical Pharmacology, College of Pharmacy, Harbin Medical University, Harbin, China; ^4^ Translational Medicine Research and Cooperation Center of Northern China, Heilongjiang Academy of Medical Sciences, Harbin, China

**Keywords:** ischemia–reperfusion injury, cardiomyocyte, m6A modification, Mettl14, Wnt/β-catenin

## Abstract

N6-methyladenosine (m6A) methylation in RNA is a dynamic and reversible modification regulated by methyltransferases and demethylases, which has been reported to participate in many pathological processes of various diseases, including cardiac disorders. This study was designed to investigate an m6A writer Mettl14 on cardiac ischemia–reperfusion (I/R) injury and uncover the underlying mechanism. The m6A and Mettl14 protein levels were increased in I/R hearts and neonatal mouse cardiomyocytes upon oxidative stress. Mettl14 knockout (Mettl14^+/−^) mice showed pronounced increases in cardiac infarct size and LDH release and aggravation in cardiac dysfunction post-I/R. Conversely, adenovirus-mediated overexpression of Mettl14 markedly reduced infarct size and apoptosis and improved cardiac function during I/R injury. Silencing of Mettl14 alone significantly caused a decrease in cell viability and an increase in LDH release and further exacerbated these effects in the presence of H_2_O_2_, while overexpression of Mettl14 ameliorated cardiomyocyte injury *in vitro*. Mettl14 resulted in enhanced levels of Wnt1 m6A modification and Wnt1 protein but not its transcript level. Furthermore, Mettl14 overexpression blocked I/R-induced downregulation of Wnt1 and β-catenin proteins, whereas Mettl14^+/−^ hearts exhibited the opposite results. Knockdown of Wnt1 abrogated Mettl14-mediated upregulation of β-catenin and protection against injury upon H_2_O_2_. Our study demonstrates that Mettl14 attenuates cardiac I/R injury by activating Wnt/β-catenin in an m6A-dependent manner, providing a novel therapeutic target for ischemic heart disease.

## Introduction

Acute myocardial infarction (AMI) is one of the leading causes of death worldwide. The most effective strategies to improve the clinical outcome of AMI patients are early and timely reperfusion therapy including thrombolysis, angioplasty, or bypass surgery by percutaneous coronary intervention. However, reperfusion itself can also cause damage to the MI heart, indicative of enhanced myocardial infarct size and cell death—a phenomenon termed ischemia–reperfusion (I/R) injury (Gul-Kahraman et al., 2019). The expansion of the infarct area due to I/R injury will continually contribute to progressive cardiac remodeling, heart failure, and even sudden cardiac death. Despite the beneficial effects in the attenuation of I/R injury obtained by many pharmacological interventions, the clinical trials have still been disappointing (Krzywonos-Zawadzka et al., 2017; Han et al., 2019). A better understanding of I/R pathogenesis is therefore urgently required to develop the novel therapeutic approach.

The posttranscriptional regulation of gene expression is critical for altering protein levels, which plays an important role in cardiac diseases progression. N6-methyladenosine (m6A) is a reversible modification found in certain classes of RNA which is dynamically controlled by methyltransferase-like 3 (Mettl3), Mettl14, WT1-associated protein (WTAP), and demethylases alpha-ketoglutarate dependent dioxygenase (FTO) and alkB homolog 5 (ALKBH5) (Roundtree et al., 2017). The recognition of methylated RNA by m6A reader proteins impacts RNA splicing, stability, and translation (Kasowitz et al., 2018; Wang and Lu, 2021). It is well established that m6A modification is involved in many biological processes in different species (Jiang et al., 2021). Recent studies have demonstrated that m6A plays a critical role in the pathophysiology of various cardiac diseases, including cardiomyocyte contractility, autophagy, fibrosis, and hypertrophy (Kumari et al., 2021). These studies consistently confirmed the elevation in m6A levels due to abnormal changes of methyltransferases and demethylases in different cardiac diseases. For example, Mettl3, as a major methyltransferase, has been well studied in the heart upon different stimuli. M6A manipulation by silencing Mettl3 expression can reduce cardiac hypertrophy, fibrosis, and autophagy in pressure overload and MI-induced cardiac remodeling (Dorn et al., 2019; Li et al., 2021; Song et al., 2019). FTO is downregulated in the failing hearts, and the overexpression of FTO preserves cardiac function by demethylating cardiac contractile transcript, thereby enhancing its stability after MI (Mathiyalagan et al., 2019). However, m6A modifications in cardiac pathologies are still incompletely understood.

The Wnt signaling pathway is a conserved and tightly controlled regulator of many physiological and pathological processes in various organ tissues through canonical and non-canonical pathways (Tewari et al., 2021). In the canonical pathway, Wnt initiates signaling cascades *via* posttranslational modification of β-catenin after binding to Frizzled family of transmembrane receptors and co-receptors (Logan and Nusse, 2004). This binding will dissociate GSK3β from molecular complexes, thereby preventing phosphorylation and subsequent of β-catenin, and cause nuclear translocation of β-catenin where it induces activation of the transcription complex T-cell factor/lymphoid enhancer factor (Doble et al., 2007). The canonical Wnt/β-catenin has been shown to play critical roles in the regulation of cardiogenesis (Moretti et al., 2006; Lin et al., 2007; Qyang et al., 2007; Kwon et al., 2009; Buikema et al., 2013), cardiac regeneration, and pathological cardiac remodeling and injury (Bergmann, 2010; Gessert and Kühl, 2010; Oerlemans et al., 2010; Duan et al., 2012; Fu et al., 2019). Wnt/β-catenin has been reported to be activated to promote cardiac fibroblast to proliferate and repair the hearts in response to acute ischemic cardiac injury (Duan et al., 2012). In addition, the activation of Wnt/β-catenin by overexpressing Wnt1 and pharmacological strategy exhibits striking protection against cell death during hepatic and kidney I/R injury in mice (Chen et al., 2015; Z. Han et al., 2020; Lehwald et al., 2011). Also, one study has revealed that the induction of Wnt/β-catenin signaling activation by the inhibition of miR-148b ameliorates cardiac I/R injury by reducing cardiomyocyte apoptosis (Yang et al., 2019). Thus, a novel mechanism regulating Wnt/β-catenin signaling is worthy of being determined.

In this study, we identified Mettl14 protein along with m6A levels was significantly increased in I/R hearts and oxidative stress–induced cardiomyocyte injury. By performing both gain- and loss-of-function approaches *in vivo* and *in vitro*, we found that Mettl14 exhibited a cardioprotective effect during I/R injury. Our findings revealed a novel mechanism by which Mettl14 attenuates cardiomyocyte injury by enhancing m6A modification of Wnt1 transcript, thereby promoting its translation, leading to activation of Wnt/β-catenin signaling.

## Materials and Methods

### Animals

Approximately 10-week-old male C57BL/6 mice weighing 18 to 22 g were provided by Changsheng Bio-Technology, Liaoning, China. Mettl14 heterozygous (+/−) mice (C57BL/6 background, 8–10 weeks) were purchased from Cyagen, Guangzhou, China. Mice were housed in a facility with a room temperature of 23 ± 2°C and a humidity of 55 ± 5%. The air exchange rate is 10–15 times/h with fresh air, and 12 h of alternating light is maintained every day. All animal procedures conformed to the Guide for the Care and Use of Laboratory Animals published by the U.S. National Institutes of Health and were approved by the Animal Ethical Committee of Harbin Medical University.

### Mouse Model of Cardiac I/R

**FIGURE 1:**
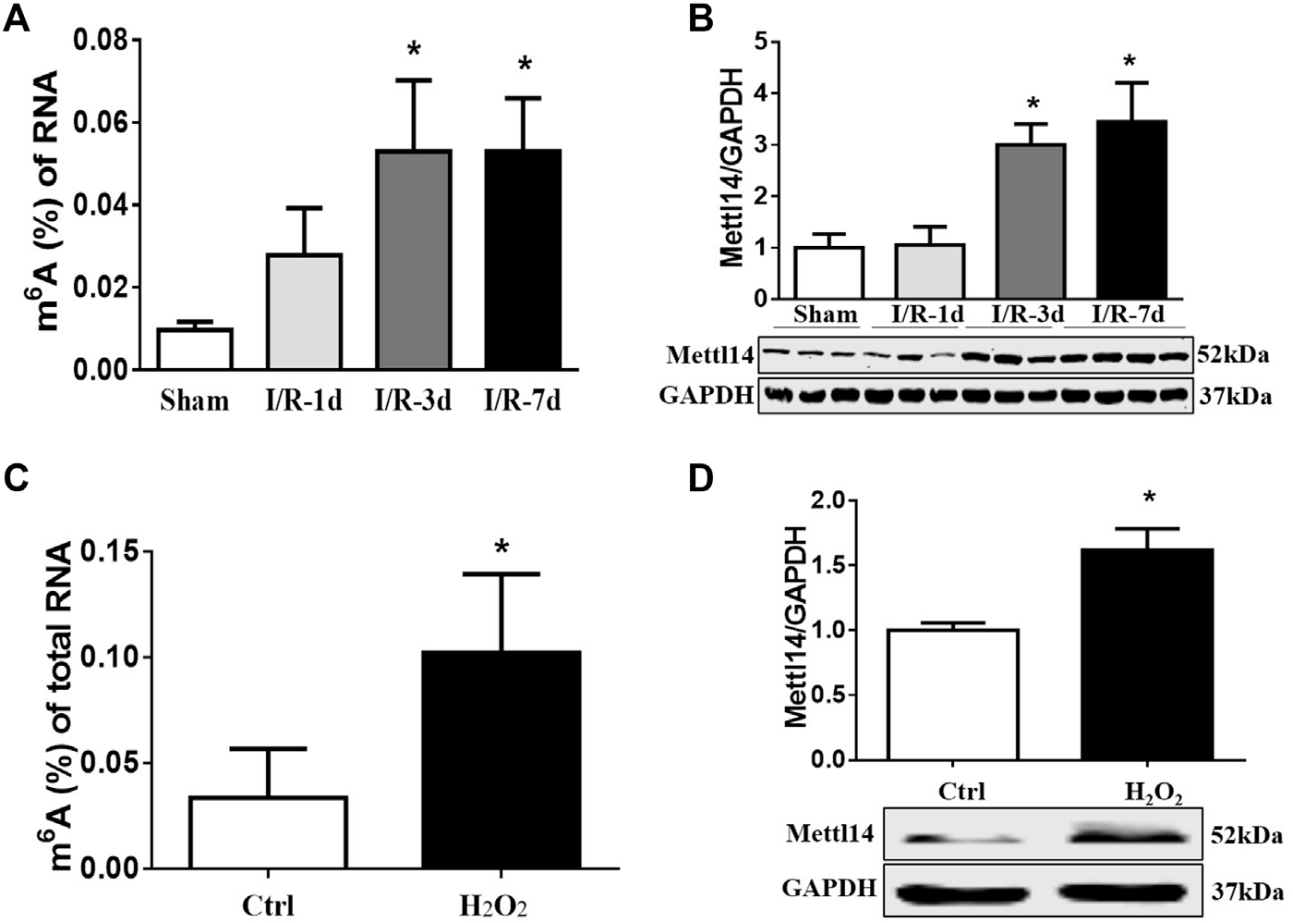
m6A modification and Mettl14 protein are elevated in heart post-I/R. **(A)** Enzyme-linked immunosorbent assay (ELISA) detects the level of m6A modification of RNA at 1, 3, and 7 days after I/R surgery in mice. Sham *n* = 5, I/R-1d *n* = 3, I/R-3d *n* = 4, I/R-7d *n* = 5. *p < 0.05 versus sham. **(B)** The protein level of Mettl14 in ischemia–reperfusion (I/R) 1, 3, and 7 days and sham-operated hearts was analyzed by Western blotting. Sham *n* = 4, I/R-1d *n* = 4, I/R-3d *n* = 4, I/R-7d *n* = 5. *p < 0.05 versus sham. **(C)** Detection of the RNA m6A modification level in cardiomyocytes treated with H_2_O_2_ (100 µM) by ELISA. Control *n* = 6, H_2_O_2_ *n* = 3. *p < 0.05 versus control. **(D)** The Mettl14 protein level in 100 µM H_2_O_2_-treated cardiomyocytes was analyzed by Western blotting. Ctrl *n* = 5, H_2_O_2_ *n* = 4.*p < 0.05 versus Ctrl. Data are represented as mean ± SEM.

**FIGURE 2:**
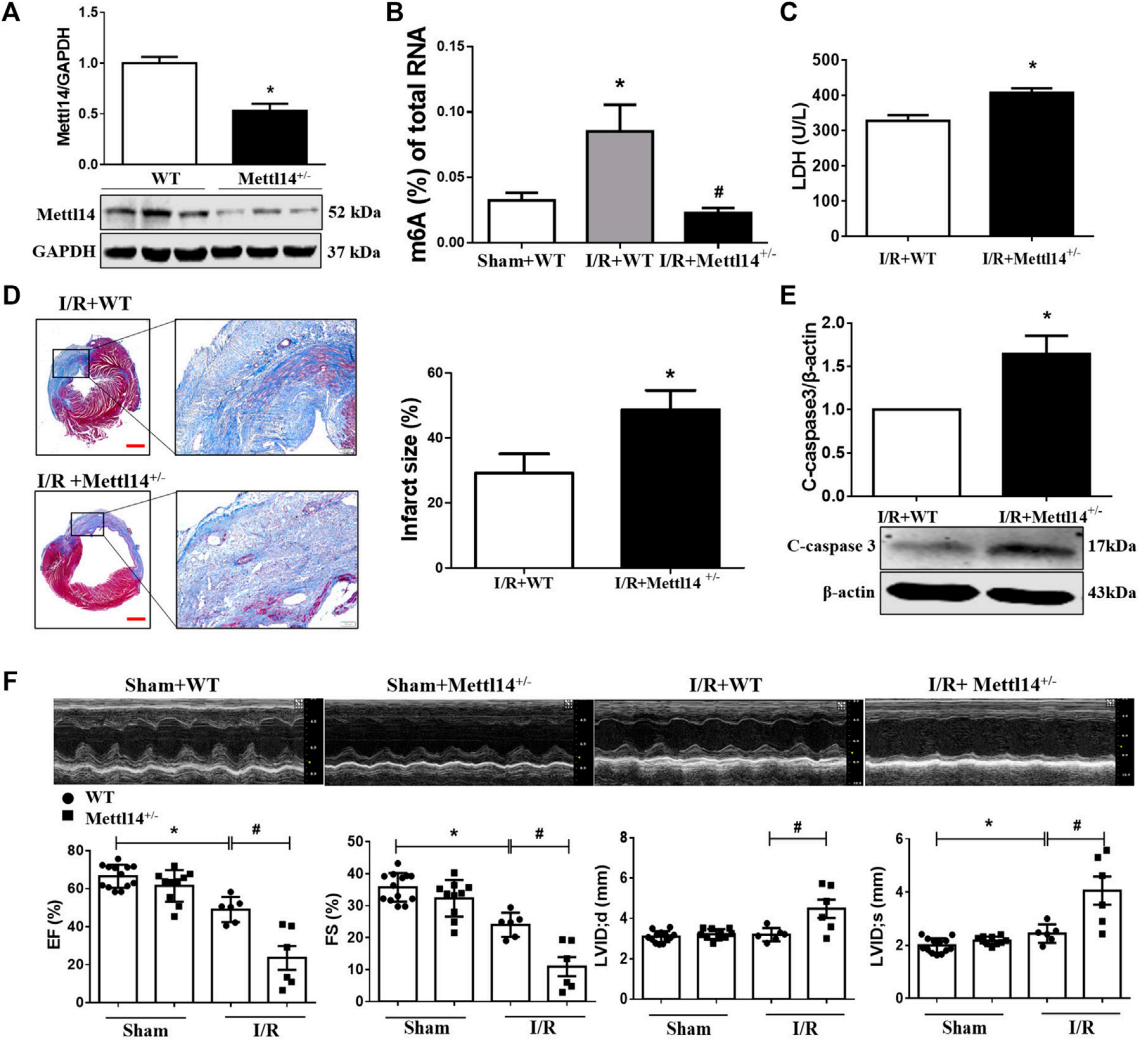
Deficiency of Mettl14 aggravates cardiac injury and dysfunction in I/R mice. **(A)** Verification of Mettl14 knockdown efficiency. The knockdown mice were subjected to sham or I/R operation for 7 days. Mettl14 protein levels in Mettl14^+/−^ mice were analyzed by Western blotting. *n* = 6 mice per group. *p < 0.05 versus WT. **(B)** Enzyme-linked immunosorbent assay (ELISA) detects the level of m6A modification of total RNA. Sham+WT *n* = 5, I/R+WT *n* = 4, I/R+Mettl14^+/−^ *n* = 4. *p < 0.05 versus sham+WT. ^#^ *p* < 0.05 versus I/R+WT. **(C)** Plasma was obtained from mice at 4 weeks of I/R. Plasma LDH activity was analyzed by using a lactate dehydrogenase assay kit. *n* = 3 mice per group. *p < 0.05 versus I/R+WT. **(D)** Infarct scar of I/R-7 day; sham-operated mice was treated by Masson’s trichrome, scale bar = 500 µm **(left),** scar bar = 100 µm **(right).**The midline length measurement was used to determine infarct scar. *n* = 5 mice per group. *p < 0.05 versus I/R+WT. **(E)** The cleaved caspase-3 expression was analyzed by Western blotting. *n* = 4 mice per group. *p < 0.05 versus I/R+WT. **(F)** Representative images of echocardiographs and statistics of ejection fraction (EF), fractional shortening (FS), left ventricular internal dimension at systole (LVIDs), and left ventricular internal dimension at end-diastole (LVIDd). Sham+WT *n* = 13, sham+Mettl14^+/−^ *n* = 10, I/R+WT *n* = 6, I/R+Mettl14^+/−^ *n* = 6. *p < 0.05 versus sham+WT. ^#^ *p* < 0.05 versus I/R+WT. Data are represented as mean ± SEM.

C57BL/6 mouse hearts were subjected to ischemia/reperfusion (I/R) *in vivo* as described previously (Bock-Marquette et al., 2004; Song et al., 2015; Brocard et al., 2017). I/R injury in mice was induced by 45-min ischemia, followed by 7-day and 4-week reperfusion in a loss-of-function study (Figure 1) and gain-of-function study (Figure 2), respectively. In brief, mice were anesthetized with 2% avertin (0.1 ml/10g body weight; Sigma-Aldrich Corporation, United States) through intraperitoneal injection. To generate I/R injury, the left anterior descending coronary artery (LAD) was ligated with 7–0 nylon for 45 min and then was removed. For the sham group, a suture was passed under the LAD but without ligation. According to the experimental requirements, at different time points of cardiac I/R, the mice were anesthetized for assessing heart function by echocardiographic measurement. All the mice survived during the process of I/R injury after the operation. Mouse hearts were isolated rapidly, and tissue samples from the ischemic area were harvested and stored at −80°C for further assays.

### Generation of Mettl14^+/−^ KO Mice

Mettl14 heterozygous (+/−) mice (C57BL/6 background) were purchased from Cyagen (Guangzhou, Guangdong, China). Mettl14^+/−^ mouse line was generated by the CRISPR/Cas9-mediated genome engineering. In brief, 11 exons were identified, and exons 7–10 were selected as the target site. Cas9 mRNA and gRNA generated by *in vitro* transcription were then injected into the fertilized eggs for Mettl14 knockout production. The targeted region of the Mettle14 gene was gRNA1 (matching the forward strand of the gene): ATT​ATT​ACT​GGG​TGG​TGT​ACA​GG; gRNA2 (matching the reverse strand of the gene): TGC​CTG​TGT​ATA​GTA​ACG​TCA​GG. The schematic targeting strategy for Mettl14^+/−^ is depicted in Supplementary Figure S2A. The founders were genotyped by PCR, followed by DNA sequencing analysis, and the positive founders were bred to the next generation that was further verified by PCR genotyping and DNA sequencing analysis. All mice were only compared to wild-type gender-matched littermates and were about 8–10 weeks old.

### Adenovirus Injection

Mettl14-carrying adenovirus for overexpression (Adv-Mettl14) and empty vector-carrying adenovirus (Adv-Null) were constructed by Cyagen (Guangzhou, China). C57BL/6 male mice were randomly selected and given Adv-Mettl14 (with 1.0 × 10^11^vector genomes per mouse) *via* tail vein injection to overexpress Mettl14 in mice. After 3 days, mice were treated for I/R or sham operation.

### Echocardiographic Analysis

Echocardiography was used to assess left ventricular (LV) function using a Vevo2100 echocardiographic system (VisualSonics, Toronto, ON, Canada) at a probe frequency of 10 MHz. Mice were anesthetized with avertin, allowing for non-invasive examination. The LV internal dimension at end-diastole (LVIDd) and the LV internal dimension at systole (LVIDs) were measured at the maximal and minimal diameters, respectively. Ejection fraction (EF%) and fractional shortening (FS%) were detected by M-mode tracings and based on statistical analysis on an average of three cardiac cycles.

### Infarct Scar Measurement

The mid-papillary slice of the LV was collected by surgery, followed by fixing with 4% paraformaldehyde (Biosharp, Hefei, China) and embedding in paraffin. The embedded LVs were cut into 6-mm slices by a paraffin sectioning machine (Thermo Fisher Scientific, Waltham, MA, United States) and fixed on adhesive slides for later use. A Masson’s Trichrome Staining kit (Solarbio, Beijing, China) was used to determine infarct size, as described previously (Takagawa et al., 2007; Yurista et al., 2019). The entire LV was captured using image analysis (FV300, Olympus, Japan). The infarct size was quantified by midline length measurement (Takagawa et al., 2007) using ImageJ software (National Institutes of Health, Bethesda, MD, United States).

### Neonatal Mouse Ventricular Cardiomyocytes Isolation and Culture

Cardiomyocytes (CMs) from 1- to 3-day-old neonatalmice were isolated using 0.25% trypsin (Solarbio, Beijing, China). The isolated hearts were washed and minced in Dulbecco’s modified Eagle medium (DMEM) (Biological Industries, Kibbutz Beit Haemek, Israel). Then the tissue block was dispersed in 0.25% pancreatin (Solarbio, Beijing, China), the pancreatin lysate was collected, and 10% fetal bovine serum (10% FBS) (Biological Industries, Haemek, Israel) and 1% penicillin/streptomycin (Beyotime, Shanghai, China) were added to DMEM medium to terminate the digestion. After centrifugation at 1500 × *g* for 5 min, the cells were resuspended in DMEM containing FBS and antibiotics and cultured in a 37°C incubator with 5% CO_2_. After 90 min, the cell suspension was seeded in a 6-well plate at a density of 1 × 10^6^ cells per well. 5-Bromo-2-deoxyuridine (10 nM) was added to remove fibroblasts.

### Cell Transfection and Treatment

Mettl14-specific siRNA (si-Mettl14), Wnt1-specific siRNA (siWnt1), and a negative control siRNA (si-NC) were purchased from Ribobio (Guangzhou, Guangdong, China). The sequences of si-Mettl14 were sense 5′-GCAGCACCUCGGUCAUUUAdTdT-3′ and antisense 5′-UAAAUGACCGAGGUGCUGCdTdT-3′. The sequences of si-Wnt1-1 were sense 5′-GCUGUGCGAGAGUGCAAAUdTdT-3′ and antisense 5′-CGA​CAC​GCU​CUC​AUG​UUU​A dTdT-3′. The sequences of si-Wnt1-2 were sense 5′-GCGUUCAUCUUCGCAAUCAdTdT-3′ and antisense 5′-UGAUUGCGAAGAUGAACGCdTdT-3′. The sequences of si-Wnt1-3 were 5′-CCUCGUCUACUUCGAGAAAdTdT-3′ and antisense 5′-GGA​GCA​GAU​GAA​GCU​CUU​U dTdT-3′. siRNAs were transfected with neonatal CMs at a final concentration of 50 nM using X-treme GENEene Transfection Reagent (Roche, Basle, Switzerland) according to the manufacturer’s instructions for 48 h. In addition, neonatal CMs were transfected by adding adenoviruses expressing green fluorescent protein (Adv-Null, viral titer 1.0 × 10^12^ PFU/ml) or GFP-fused Mettl14 (Adv-Mettl14, viral titer 1.0 × 10^12^ PFU/ml) for 48 h. After 24 h, cells were then exposed to H_2_O_2_, with a final concentration of 100 µM for 24 h.

### Hoechst 33342 and Propidium Iodide (PI) Fluorescent Staining

Hoechst 33342 and propidium iodide (PI) fluorescent staining were performed as previously described (Li et al., 2020). The neonatal CMs were seeded in 24-well plates at a density of 1.0 × 10^5^/well. According to the experimental requirements, neonatal CMs were incubated at 4°C for 20 min under dark conditions with Hoechst 33342 and PI (Solarbio, Beijing, China), respectively. The fluorescence signal was detected by a confocal laser scanning microscope (FV300, Olympus, Japan).

### Cell Viability by CCK8 Assay

Neonatal CMs were cultured in 96-well plates at a number of 6 × 10^4^ per well, followed by a CCK8 assay to measure cell viability. After various treatments according to the experimental requirements, the medium of each well was replaced with 100 µl medium containing 10 µl of CCK8 solution, and then the cells were incubated again for 1.5–2 h at 37°C in the dark. Finally, the absorbance was measured at 450 nm in a microplate reader.

### Lactate Dehydrogenase (LDH) Release Assay

Cardiomyocytes were cultured in six-well plates at a density of 1 × 10^6^ cells per well. According to experimental requirements, the culture medium samples were collected. The concentration of lactate dehydrogenase (LDH) was detected following the manufacturer’s instructions of a lactate dehydrogenase assay kit (Nanjing Jiancheng, Jiangsu, China).

### Western Blotting

Western blot analysis was performed as previously described (Li et al., 2020). Total tissue/cell proteins were extracted using a lysis buffer (Roche, Switzerland) containing 1% protease inhibitor and 10% phosphatase inhibitor. Protein concentration was determined by using a bicinchoninic acid (BCA) protein kit (Beyotime Institute of Biotechnology, Shanghai, China) incubation. Different concentrations of sodium dodecyl sulfate–polyacrylamide gel electrophoresis (SDS-PAGE) were prepared for equivalent protein electrophoresis and nitrocellulose membrane transfer. Next, the membrane was incubated overnight with anti-Mettl14 (#ab98166, 1:1000; Abcam, Cambridge, United Kingdom), anti-FTO (#ab92821, 1:1000; Abcam, Cambridge, United Kingdom), anti-Mettl4 (#ab107540, 1:1000; Abcam, Cambridge, United Kingdom), anti-ALKBH5 (#NBP1-82188, 1:1000; Novusbio, CO, United States), anti-WTAP (#sc-374280, 1:500; Santa Cruz, DE, United States), anti-Mettl3 (#ab98166, 1:1000; Abcam, Cambridge, United Kingdom), anti-GAPDH (#TA-08, 1:1000; ZsBio, Beijing, China), anti-β-catenin (#8480, 1:1000; Cell Signaling Technology, MA, United States), anti-caspase-3 (#9662, 1:750; Cell Signaling Technology, MA, United States), anti-Wnt1 (#GTX105955, 1:1000; Gene Tex, TX, United States), and anti-β-tubulin (#AC021, 1:5000; ABclonal, Wuhan, China) in a 4°C refrigerator. After Tris-buffered saline and Tween 20 (TBST) washing, the membranes were incubated with the secondary antibody at room temperature for 60 min. An Odyssey infrared imaging system (LI-COR, Lincoln, NE, United States) was used to quantify the band intensity and measure the gray value.

### Real-Time Quantitative Reverse Transcriptase-PCR (qRT-PCR)

Total RNA was extracted from collected cells or tissues by using TRIzol reagent (Invitrogen, Carlsbad, CA, United States). The concentration of RNA samples was detected by NanoDrop ND-8000 (Thermo Fisher Scientific, Waltham, MA, United States). cDNA was obtained by reverse transcription using a reverse transcription kit (Toyobo, Japan). SYBR Green (Toyobo, Japan) was used in real-time PCR assays to quantify Dvl1, Mettl14, Axin2, Ccnd1, Ccnd2, and Wnt1 mRNA levels on a 7500 FAST Real-Time PCR System (Applied Biosystems, Foster City, CA, United States). Normalized RNA expression was calculated using the comparative cycle threshold (Ct) method (2^−ΔΔCt^). Gene expressions were normalized to GAPDH in each sample. All primers used in this study were Axin2-F 5′-TAC​CTC​CCC​ACC​TTG​AAT​GA-3′ and Axin2-R 5′-TTG​ACT​GGG​TCG​CTT​CTC​TT-3′, Dvl1-F 5′-CGG​AGC​TAC​TTC​ACC​ATC​CC-3′ and Dvl1-R 5′-CAC​TCT​TCA​CAG​TCA​GCG​GT-3′, Ccnd2-F 5′-CGT​GTT​CGT​CAT​CTG​CTA​GC-3′ and Ccnd2-R 5′-CAG​GAC​TTT​GAA​CAG​GCA​CC-3′, Mettl14-F 5′-TCT​GGA​AAA​CTG​CCT​TTG​GAT-3′ and Mettl14-R 5′-AAA​TGC​TGG​ACC​TGG​GAT​GAT-3′, Dvl1-F 5′-CGG​AGC​TAC​TTC​ACC​ATC​CC-3′ and Dvl1-R 5′-CAC​TCT​TCA​CAG​TCA​GCG​GT-3′, Ccnd1-F 5′-AGA​AGT​GCG​AAG​AGG​AGG​TC-3′ and Ccnd1-R 5′-TTC​TCG​GCA​GTC​AAG​GGA​AT-3′ and Wnt1-F 5′-CGA​CTG​ATC​CGA​CAG​AAC​CC-3′ and Wnt1-R 5′-CCA​TTT​GCA​CTC​TCG​CAC​A-3′, GAPDH-F 5′-AAG​AAG​GTG​GTG​AAG​CAG​GC-3′, and GAPDH-R 5′-TCC​ACC​ACC​CTG​TTG​CTG​TA-3′.

### Quantification of m6A Quantification

Total RNA was isolated by using TRIzol agent (15596018; Invitrogen) following the manufacturer’s instructions. The concentration of RNA was measured by a NanoDrop. The m6A levels in total RNA were quantified by an m6A RNA methylation assay kit (Cat#ab185912; Abcam, Cambridge, United Kingdom) according to the manufacture’s protocol.

$$m6A\%=\frac{Sample_{OD}-NC_{OD}}{S\left(PC_{OD}-NC_{OD}\right)}÷\text{P}\times 100\text{\%,}$$

where S represents the amount of input RNA sample (ng), P is the amount of input of positive control (ng), and PC represents the positive control.

### Microarray Analysis

Total RNA samples were extracted from Adv-Mettl14–infected cardiomyocytes and the corresponding non-target control cells. mRNA microarray analysis was performed by Arraystar company (Rockville, MD, United States). In brief, the total RNAs were immunoprecipitated with an anti-N6-methyladenosine (m6A) antibody. The elution from the immunoprecipitation magnetic beads was called “IP.” The recovered supernatant was called “Sup,” and labels “IP” and “Sup” RNA were used for Cy5 and Cy3, respectively. After merging, it was hybridized to Arraystar Human m6A Epitranscriptomic Microarray (8 × 60 K, Arraystar). Finally, an Agilent scanner G2505C was used to scan the array.

### Methylated RNA Immunoprecipitation (MeRIP)-qRT-PCR

The level of m6A modification of genes was measured by using a Magna MeRIP Kit (Millipore, cat. CR203146) according to the manufacturer’s instructions. In brief, the cells were collected and suspended in RIP lysis buffer. The pyrolysis products can be stably stored at −80°C for 3 months. M6A antibody (5 μg) (Synaptic System No. 202003) or IgG antibody was added to a tube containing magnetic beads, followed by rotation at RT for 30 min. The beads were washed with RIP wash buffer twice and resuspended in 900 μl of RIP buffer mixed with 100 μl of cell lysate, followed by centrifugation at 14,000 rpm at 4°C for 10 min. After rotation at 4°C overnight, the beads were washed with a high-salt buffer, followed by extraction of RNAs with RIP wash buffer. Finally, the RNA was extracted and analyzed by qRT-PCR.

### Statistical Analysis

All values are presented as means ± standard error of the mean (SEM). Student’s unpaired two-tailed *t*-test was used for two-group comparisons, while one-way analysis of variance (ANOVA) followed by Tukey’s *post hoc* correction was used for multigroup comparisons. GraphPad Prism 7.0 software (GraphPad Software, San Diego, CA, United States) was used for statistical analyses. A *p*-value <0.05 was considered statistically significant.

## Results

### Upregulation of Mettl14 in Heart Post-I/R

To uncover RNA m6A methylation may participate in cardiac I/R injury, we constructed a mouse model of cardiac ischemia–reperfusion (I/R) over time and determined the expression levels of m6A methylation and related methyltransferases and demethylases in the peri-infarct zone of cardiac tissue. As expected, cardiac function was compromised as reflected by reduced fractional shortening in mice subjected to I/R (Supplementary Figure S1A). We observed a slight increase in the m6A level in total RNA at day 1 and persistent upregulation within 1 week in the infarcted hearts post-I/R as compared with sham-operated animals (Figure 1A). Western blot data revealed that Mettl14 and FTO protein levels exhibited the most striking changes with the opposite trends after I/R. The upregulation of the Mettl14 protein level in the mouse heart upon I/R may explain the elevated m6A levels in impaired hearts (Figure 1B and Supplementary Figures S1B–D). To verify the involvement of cardiomyocytes in causing increases in Mettl14 and m6A levels in I/R mice, we established an *in vitro* model of oxidative stress in cultured neonatal mouse cardiomyocytes (neonatal CMs) *via* treatment with H_2_O_2_. In line with *in vivo* data, Mettl14 protein and m6A levels were markedly increased in neonatal CMs after exposure to H_2_O_2_ (Figures 1C,D). These results suggest Mettl14-dependent m6A catalysis in cardiomyocytes may be implicated in the pathological process of cardiac I/R injury.

### Deficiency of Mettl14 Aggravates Cardiac Injury and Dysfunction in I/R Mice

To determine the functional role of Mettl14 in cardiac I/R, we generated the Mettl14 knockout (Mettl14^+/−^) mice model using the CRISPR/Cas9 system (Supplemental Figure S2A). A significantly reduced level in Mettl14 protein was shown in the heart tissue of Mettl14^+/−^ mice as compared to their wild-type (WT) littermates (Figure 2A). This reduction in cardiac Mettl14 protein expression markedly abolished the elevated m6A level of total RNA in I/R-treated mice (Figure 2B). To exclude the effect of Mettl14 knockdown on other components of the m6A-catalyzing complex, we assessed the protein expression of m6A methyltransferases and demethylases in Mettl14^+/−^ hearts. Of these, only the cardiac WTAP protein level was changed but not for others in Mettl14^+/−^ mice (Supplementary Figure S2B). To evaluate whether endogenous Mettl14 was required to protect against cardiac injury under a pathological context, we subjected Mettl14^+/−^ mice to I/R surgery. The serum level of LDH, an indicator of cardiac damage, released from I/R hearts was further increased in Mettl14^+/−^ mice (Figure 2C). Notably, the deficiency of Mettl14 significantly enlarged myocardial infarct size in I/R-treated hearts compared with WT mice (Figure 2D). Furthermore, Mettl14^+/−^ hearts displayed enhanced expression in cleaved caspase-3 protein compared to WT animals upon I/R (Figure 2E), suggesting Mettl14 deletion accelerates I/R-induced apoptosis. Echocardiography analyses showed that cardiac function at the baseline level was comparable between Mettl14^+/−^ and WT mice (Figure 2F). Notably, we found that the deficiency of Mettl14 dramatically exacerbated cardiac dysfunction as indicated by reduced percent ejection fraction (EF%) and fractional shortening (FS%) in I/R-treated hearts compared with WT animals (Figure 2F). Moreover, robust increases in the left ventricular internal dimension at end-diastole (LVIDd) and end-systole (LVIDs) were displayed in Mettl14^+/−^-I/R hearts relative to the WT-I/R group (Figure 2F). These results suggest Mettl14 was indispensable to be resistant to cardiac injury in response to I/R.

### Mettl14 Attenuates Myocardial Injury Upon I/R

**FIGURE 3:**
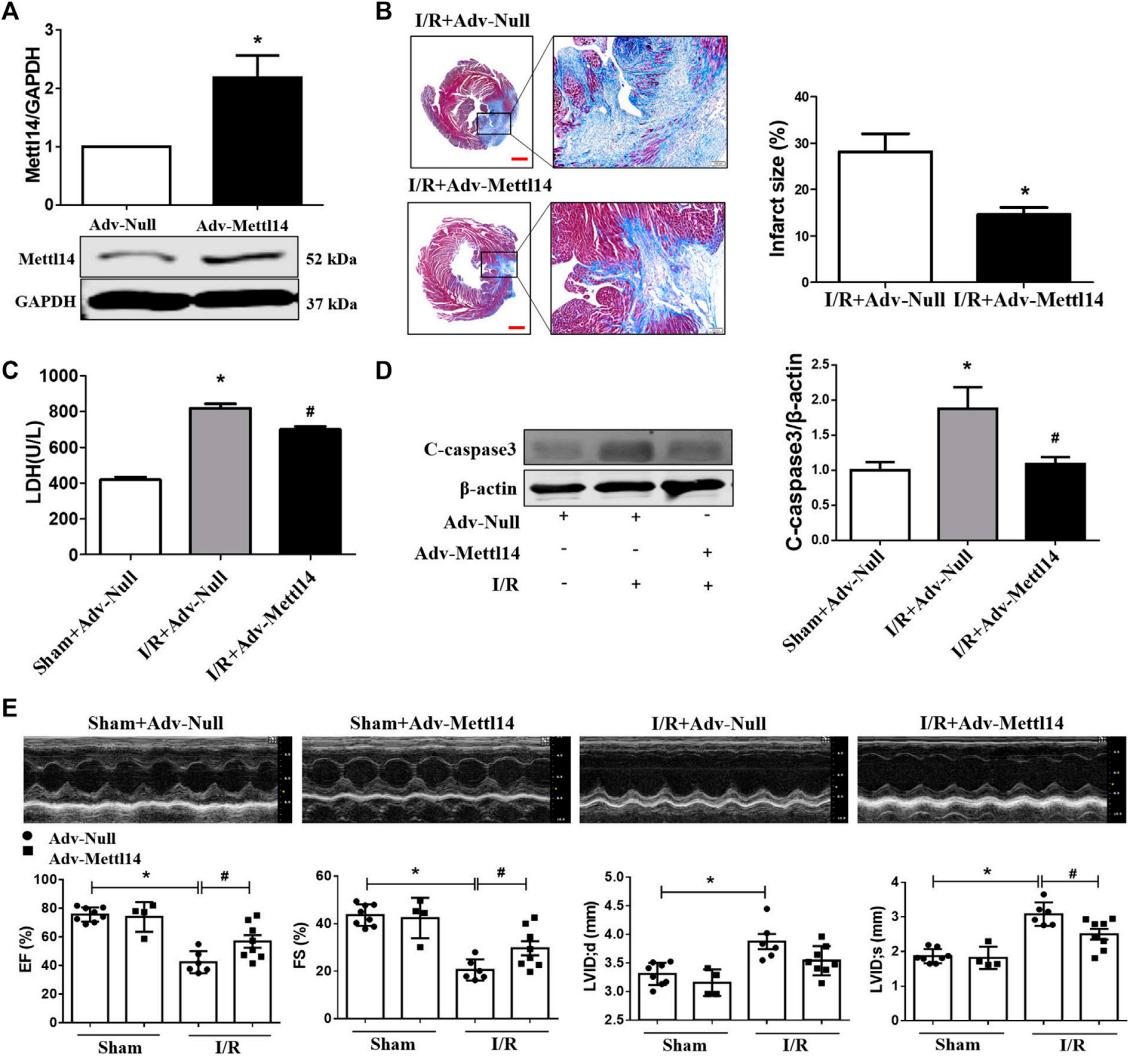
Mettl14 attenuates myocardial injury upon I/R. **(A)** Mouse hearts were infected by adenovirus. The infected mice were treated to I/R or sham operation for 4 weeks. The expression of Mettl14 was analyzed by Western blotting. Adv-Null *n* = 3, Adv-Mettl14 *n* = 4. *p < 0.05 versus Adv-Null. **(B)** Infarct scar of I/R-4 week hearts was treated by Masson’s trichrome, bar = 500 µm **(left),** scar bar = 100 µm **(right).** Midline length measurement (Takagawa et al., 2007) was used to determine infarct scar. *n* = 5 mice per group. *p < 0.05 versus I/R+Adv-Null. **(C)** Plasma LDH activity was analyzed by lactate dehydrogenase assay kit. Sham+Adv-Null *n* = 4, I/R+Adv-Null *n* = 4, I/R+Adv-Mettl14 *n* = 3. *p < 0.05 versus sham+Adv-Null. ^#^ *p* < 0.05 versus I/R+Adv-Null. **(D)** The cleaved caspase-3 expression was analyzed by Western blotting. *n* = 4 mice per group. *p < 0.05 versus Sham+Adv-Null. ^#^ *p* < 0.05 versus I/R+Adv-Null. **(E)** Representative images of echocardiographs and statistics of ejection fraction (EF), fractional shortening (FS), left ventricular internal dimension at systole (LVIDs), and left ventricular internal dimension at end-diastole (LVIDd). Sham+Adv-Null *n* = 8, sham+Adv-Mettl14 *n* = 4, I/R+Adv-Null *n* = 6, I/R+Adv-Mettl14 *n* = 8. *p < 0.05 versus sham+Adv-Null. ^#^ *p* < 0.05 versus I/R+Adv-Null. Data are represented as mean ± SEM.

A question we asked was whether the overexpression of Mettl14 could elicit cardioprotective effects upon I/R. To answer this question, we carried out a gain-of-function strategy by constructing an adenovirus vector carrying the Mettl14 gene (Adv-Mettl14) to overexpress it in mice hearts. As shown in Figure 3A, delivery of Adv-Mettl14 remarkably increased cardiac Mettl14 protein expression. The increase in Mettl14 level led to reduced myocardial infarct size in I/R mice compared with the negative control (gene-free empty virus for Mettl14, Adv-Null)–treated hearts post-I/R (Figure 3B). In addition, I/R-induced elevation in LDH levels in mouse plasma was attenuated by the overexpression of Mettl14 (Figure 3C). The cardiac cleaved caspase-3 protein level was significantly increased after I/R, which was reduced by overexpressing Mettl14 (Figure 3D). Echocardiography results showed that the overexpression of Mettl14 in sham-operated hearts failed to affect cardiac function at baseline levels, while it ameliorated cardiac dysfunction caused by I/R, as reflected by increases in LVEF and LVFS. Furthermore, we observed a significant decline in LVIDs in Mettl14-overexpressing I/R mice hearts compared with Adv-Null–treated hearts, suggesting Mettl14 reduced cardiac dilatation (Figure 3E). These data demonstrate that Mettl14 protects against myocardial injury and cardiac dysfunction upon I/R.

### Mettl14 Reduces Cardiomyocyte Injury Upon H_2_O_2_ *In Vitro*

**FIGURE 4:**
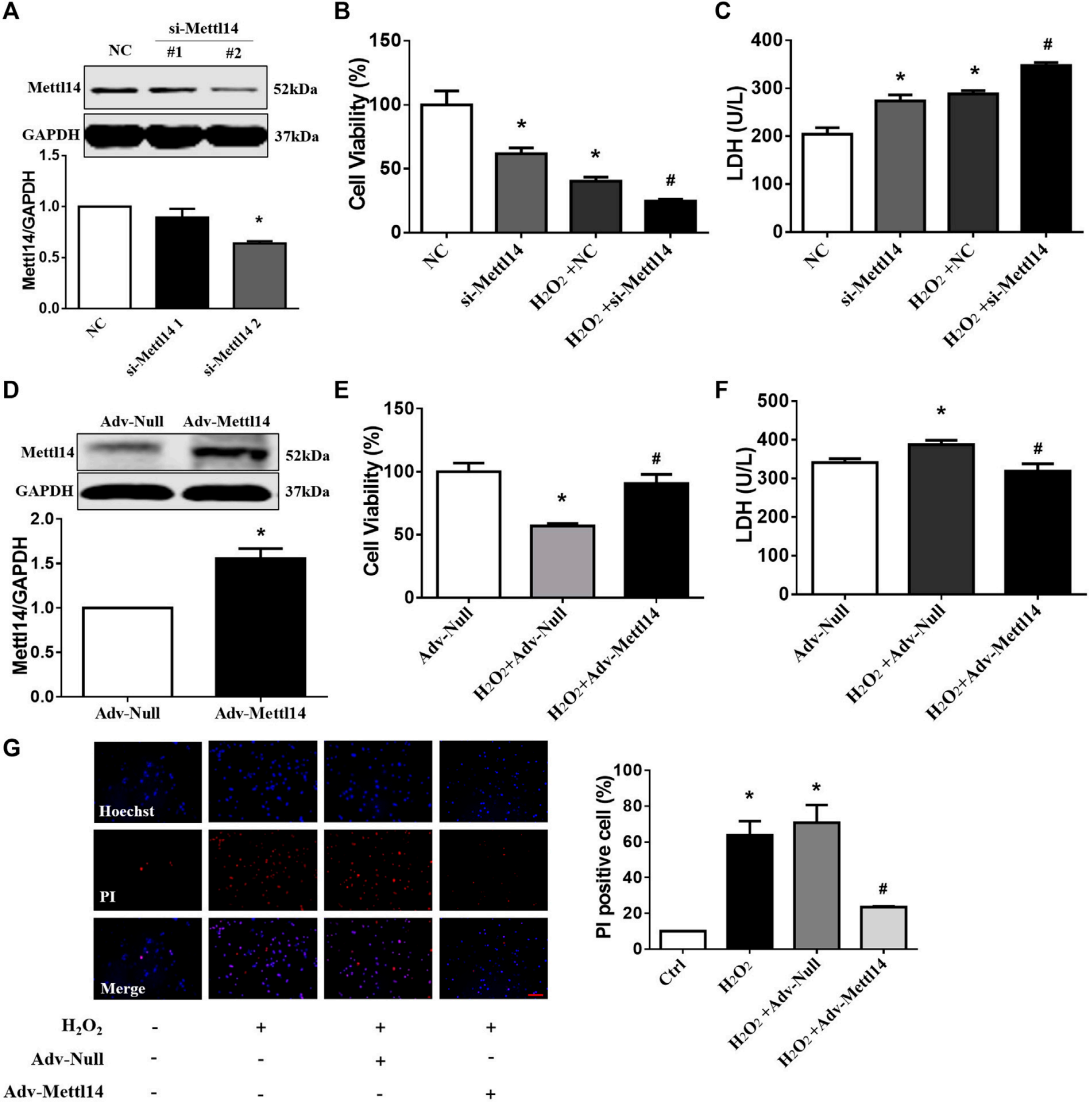
Mettl14 reduces cardiomyocyte injury upon H_2_O_2_ *in vitro*_._ **(A)** Cardiomyocytes were transfected with si-RNA and were treated with 100 µM H_2_O_2_ for 12 h. The Mettl14 protein level in cardiomyocytes was analyzed by Western blotting. *n* = 3 from three independent cultures. *p < 0.05 versus NC. **(B)** Cell viability was detected with CCK8. NC *n* = 5, si-Mettl14 *n* = 5, H_2_O_2_+NC *n* = 6, H_2_O_2_+si-Mettl14 *n* = 6. *p < 0.05 versus NC. ^#^ *p* < 0.05 versus H_2_O_2_+NC. **(C)** Culture medium activity of LDH was analyzed by using a lactate dehydrogenase assay kit. *n* = 3 from three independent cultures. *p < 0.05 versus NC. ^#^ *p* < 0.05 versus H_2_O_2_+NC. **(D**–**F)** Cardiomyocytes were infected with Mettl14 expression vector or empty vector and were treated with 100 µM H_2_O_2_ for 12 h. **(D)** The Mettl14 protein level in cardiomyocytes overexpressed Mettl14. *n* = 4 from three independent cultures. *p < 0.05 versus Adv-Null. **(E)** Cell viability was detected with CCK8. *n* = 6 from three independent cultures. *p < 0.05 versus Adv-Null. ^#^ *p* < 0.05 versus H_2_O_2_+Adv-Null. **(F)** Culture medium activity of LDH was analyzed by using a lactate dehydrogenase assay kit. Adv-Null *n* = 3, H_2_O_2_+Adv-Null *n* = 5, H_2_O_2_+Adv-Mettl14 *n* = 5. *p < 0.05 versus Adv-Null. ^#^ *p* < 0.05 versus H_2_O_2_+Adv-Null. **(G)** PI staining of damaged cardiomyocytes (red); DAPI stains all nucleus (blue), scale bar = 100 µm *n* = 3 from three independent cultures. *p < 0.05 versus Ctrl. ^#^ *p* < 0.05 versus H_2_O_2_+Adv-Null. Data are represented as mean ± SEM.

The aforementioned results led us to consider if Mettl14 regulates cardiomyocytes injury *in vitro*. To clarify this issue, we used the gain- and loss-of-function approach in neonatal CMs. The knockdown of Mettl14 in RNA and protein levels in neonatal CMs was induced by administration of a small interfering RNA (siRNA) (Figure 4A and Supplementary Figure S3A). Notably, silencing of Mettl14 alone caused a pronounced decrease in cell viability and aggravated this reduction in neonatal CMs after exposure to H_2_O_2_ (Figure 4B). Similar results showed that the inhibition of Mettl14 promoted LDH release in neonatal CMs in the absence or presence of H_2_O_2_ (Figure 4C). Next, we sought to determine whether Mettl14 reduces cardiomyocyte injury induced by H_2_O_2_. We successfully overexpressed Mettl14 by infecting an adenoviral vector carrying the Mettl14 gene (Figure 4D and Supplementary Figure S3B). As anticipated, the overexpression of Mettl14 significantly increased cell viability and reduced LDH levels in the culture medium of neonatal CMs in response to H_2_O_2_ (Figures 4E,F). Moreover, the increased number of PI-positive cells induced by H_2_O_2_ was significantly reduced in Mettl14-overexpressing neonatal CMs (Figure 4G). Collectively, these results show that Mettl14 alleviates cardiomyocyte injury under oxidative stress.

### Mettl14 Activates the Wnt/β-Catenin Signaling Pathway in an m6A-Dependent Manner

**FIGURE 5:**
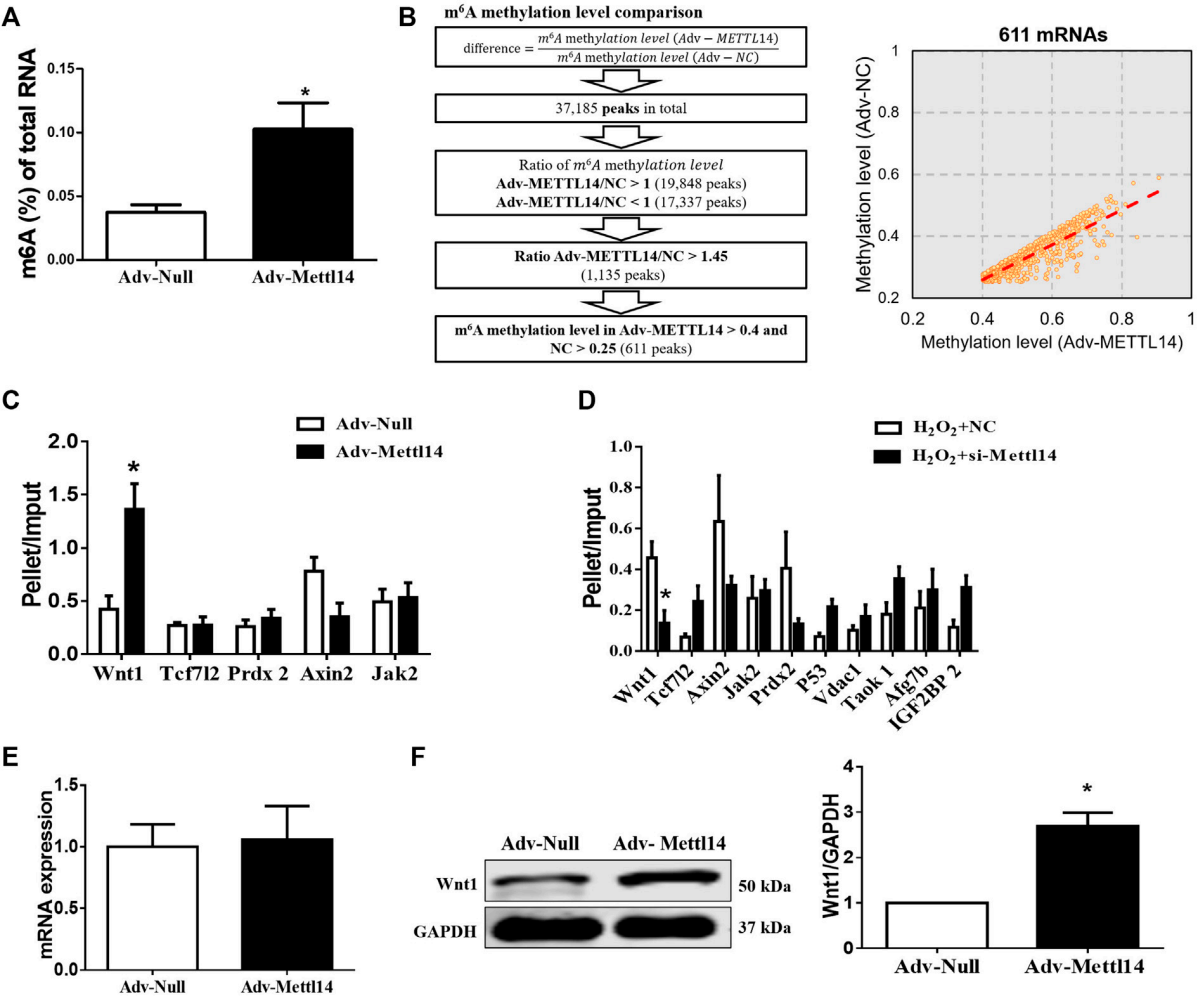
Mettl14 activates the Wnt/β-catenin signaling pathway in an m6A-dependent manner. **(A)** Enzyme-linked immunosorbent assay (ELISA) detects the level of m6A modification of RNA in cardiomyocytes treated with adenovirus. Adv-Null *n* = 4, Adv-Mettl14 *n* = 5. *p < 0.05 versus Adv-Null. **(B)** Schematic for microarray sample collection with two groups: NC, Adv-Mettl14 in cardiomyocytes, indicating 37185 genes’ different expression. *n* = 1. **(C**,**D)** m6A-immunoprecipitation (RIP) microarray examined the m6A level of mRNAs caused by Mettl14 overexpression **(C)** and silence with H_2_O_2_ **(D)**, respectively. *n* = 4 from three independent cultures. *p < 0.05 versus Adv-Null. *n* = 3 from three independent cultures. *p < 0.05 versus H_2_O_2_+NC. **(E)** Cardiomyocytes were infected with Mettl14 expression vector or empty vector. The Wnt1 mRNA level was analyzed by qRT-PCR. Adv-Null *n* = 4, Adv-Mettl14 *n* = 6. *p > 0.05 versus Adv-Null. **(F)** Wnt1 protein level in cardiomyocytes was analyzed by Western blotting. *n* = 3 from three independent cultures. *p < 0.05 versus Adv-Null. Data are represented as mean ± SEM.

To uncover the mechanism by which Mettl14 protected against cardiac I/R injury *in vivo* and *in vitro*, we performed immunoprecipitation of m6A-modified RNA (MeRIP) microarray assay in Mettl14-overexpressed cardiomyocytes to screen the m6A methylated targets of Mettl14. As expected, the overexpression of Mettl14 induced an increase in m6A levels of total RNA in neonatal CMs (Figure 5A). Our microarray results identified 37,185 differentially expressed m6A peaks, including 19,848 upregulated and 17,337 downregulated peaks in Mettl14-transfected cells compared with the NC group. We further screened 611 upregulated peaks of m6A modification by setting cutoff (FC > 1.45, Mettl14 *vs* NC) (Figure 5B). To verify the MeRIP microarray results, we employed the MeRIP-qPCR assay to quantify the expression level of 10 different m6A-catalyzed mRNAs, which are involved in the process during cardiac injury. Specifically, our results showed that among these genes, the level of m6A methylated Wnt1 mRNA was significantly increased in Mettl14-overexpressing neonatal CMs, while Mettl14 knockdown reduced its level after H_2_O_2_ treatment (Figures 5C,D). Considering m6A modification in RNA can influence mRNA stability per se and protein translation (Huang et al., 2018), we determined expression levels of Wnt1 in mRNA and protein. Notably, we found that the overexpression of Mettl14 failed to change mRNA expression of Wnt1 but remarkably increased the Wnt1 protein level in neonatal CMs (Figures 5E,F).

**FIGURE 6:**
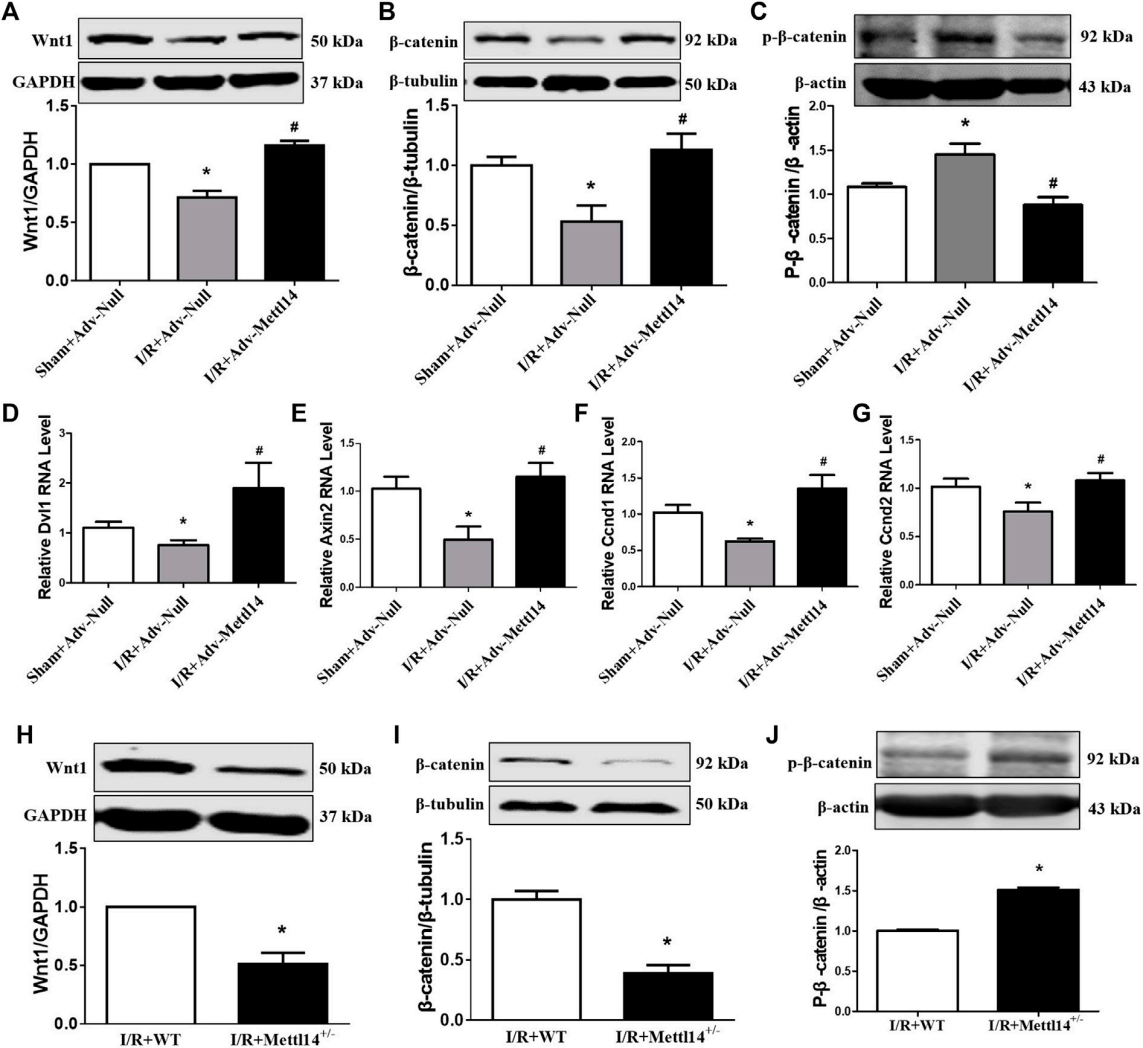
Mettl14 regulates I/R injury by activating the Wnt/β-catenin pathway. **(A**–**C)** Mouse hearts were infected by adenovirus. The infected mice were treated to I/R or sham operation. **(A)** The Wnt1 expression was analyzed by Western blotting. Sham+Adv-Null *n* = 4, I/R+Adv-Null *n* = 4, I/R+Adv-Mettl14 *n* = 3. *p < 0.05 versus sham+Adv-Null. ^#^ *p* < 0.05 versus I/R+Adv-Null. **(B)** The β-catenin expression was analyzed by Western blotting. *n* = 5 mice per group. *p < 0.05 versus sham+Adv-Null. ^#^ *p* < 0.05 versus I/R+Adv-Null. **(C)** The p-β-catenin expression was analyzed by Western blotting. *n* = 4 mice per group. *p < 0.05 versus sham+Adv-Null. ^#^ *p* < 0.05 versus I/R+Adv-Null. **(D)** The Dvl1 mRNA level was analyzed by qRT-PCR. Sham+Adv-Null *n* = 4, I/R+Adv-Null *n* = 3, I/R+Adv-Mettl14 *n* = 3. *p < 0.05 versus sham+Adv-Null. ^#^ *p* < 0.05 versus I/R+Adv-Null. **(E)** The Axin2 mRNA level was analyzed by qRT-PCR. Sham+Adv-Null *n* = 5, I/R+Adv-Null *n* = 5, I/R+Adv-Mettl14 *n* = 3. *p < 0.05 versus Sham+Adv-Null. ^#^ *p* < 0.05 versus I/R+Adv-Null. **(F)** The Ccnd1 mRNA level was analyzed by qRT-PCR. Sham+Adv-Null *n* = 5, I/R+Adv-Null *n* = 4, I/R+Adv-Mettl14 *n* = 3. *p < 0.05 versus sham+Adv-Null. ^#^ *p* < 0.05 versus I/R+Adv-Null. **(G)** The Ccnd2 mRNA level was analyzed by qRT-PCR. Sham+Adv-Null *n* = 5, I/R+Adv-Null *n* = 4, I/R+Adv-Mettl14 *n* = 3. *p < 0.05 versus sham+Adv-Null. ^#^ *p* < 0.05 versus I/R+Adv-Null. **(H**–**J)** The Mettl14 knockdown mice were subjected to sham or I/R operation. **(H)** The Wnt1 expression was analyzed by Western blotting. *p < 0.05 versus IR+WT. *n* = 5 mice per group. *p < 0.05 versus I/R+WT. **(I)** The β-catenin expression was analyzed by Western blotting. *n* = 5 mice per group. *p < 0.05 versus IR+WT. **(J)** The p-β-catenin expression was analyzed by Western blotting. *n* = 3 mice per group. *p < 0.05 versus IR+WT. Data are represented as mean ± SEM.

Existing studies have shown that the activation of the Wnt/β-catenin signaling pathway reduces myocardial I/R injury (Li et al., 2019; Yang et al., 2019). We hypothesized that Mettl14-mediated cardioprotection against I/R injury might be ascribed to Wnt/β-catenin signaling activation by regulating Wnt1 expression. In accordance with the previous studies (Jiang et al., 2020), we found that Wnt/β-catenin signaling was suppressed in response to I/R as reflected by the decreased expression level of Wnt1, β-catenin, and Dvl1 and increased the p-β-catenin protein level, which was markedly reversed by Mettl14 overexpression (Figures 6A–D). This was further verified by the results that the overexpression of Mettl14 abrogated the reduction in mRNA levels of Wnt target genes Axin2, Ccnd1, and Ccnd2 (Figures 6E–G). In sharp contrast, Mettl14^+/−^ hearts showed much lower levels of Wnt1 and β-catenin proteins and a higher level of p-β-catenin than the mice in the WT-I/R group (Figures 6H–J). The data suggest that Mettl14 regulates the activation of canonical Wnt/β-catenin signaling *in vivo*.

### Knockdown of Wnt1 abolished Mettl14-mediated protection against neonatal CM injury upon H_2_O_2_

**FIGURE 7:**
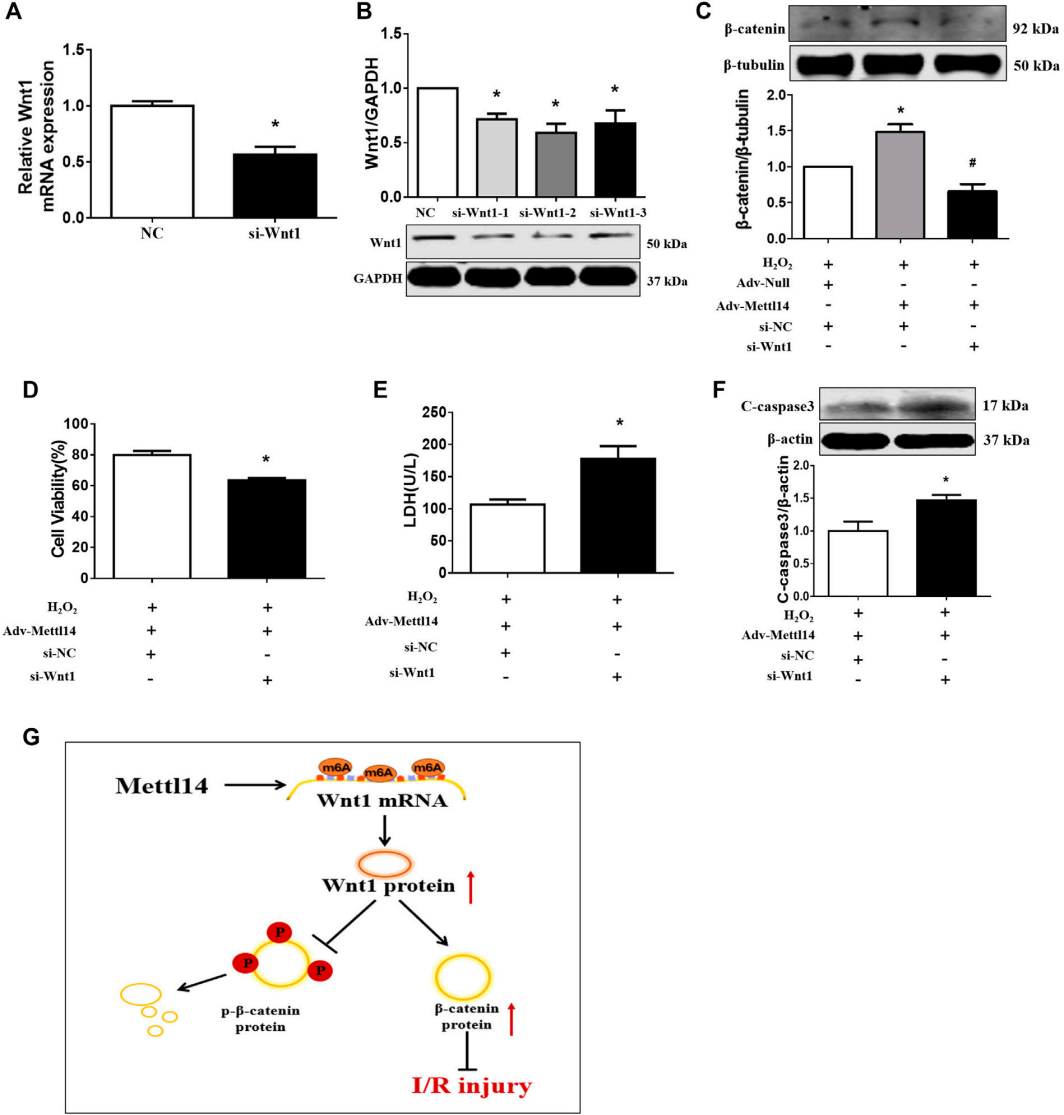
Knockdown of Wnt1 abolished Mettl14-mediated cardioprotection *in vitro*. **(A**,**B)** Cardiomyocytes were infected by si-RNA. The expression of Wnt1 was analyzed by **(A)** qRT-PCR and **(B)** Western blotting. *n* = 3 from three independent cultures. *p < 0.05 versus NC. **(C)** Cardiomyocytes were co-transfected with the Mettl14 vector or empty vector and si-Wnt1. The β-catenin protein level was analyzed by Western blotting. *n* = 3 from three independent cultures. *p < 0.05 versus H_2_O_2_+Adv-Null. ^#^ *p* < 0.05 versus H_2_O_2_+Adv-Mettl14. **(D)** Cell viability was detected with CCK8. *n* = 6 from three independent cultures. *p < 0.05 versus H_2_O_2_+Adv-Mettl14. **(E)** The culture medium level of LDH. *n* = 8 from three independent cultures. *p < 0.05 versus H_2_O_2_+Adv-Mettl14. **(F)** The cleaved caspase-3 protein level was analyzed by Western blotting. *n* = 5 from three independent cultures. *p < 0.05 versus H_2_O_2_+Adv-Mettl14. Data are represented as mean ± SEM. **(G)** Schematic showing the proposed mechanism for the effects of Mettl14 on cardiac ischemia–reperfusion injury. During cardiac ischemia–reperfusion injury, Mettl14 expression is increased, which improves Wnt1 mRNA m6A modification. Therefore, Wnt1 expression is upregulated, and the Wnt/β-catenin pathway is activated. The β-catenin phosphorylation is inhibited, and β-catenin is accumulated. To sum up, it has a protective effect on the heart damaged by ischemia–reperfusion.

We reasoned that Wnt1 might mediate the cardioprotective effects of Mettl14 in neonatal CMs. To test this notion, we sought to silence Wnt1 expression by siRNA in Mettl14-overexpressing neonatal CMs in the context of oxidative stress. Our results showed that the sequence 2 of siRNA-Wnt1 exerted most reduction of Wnt1 in mRNA and protein levels in neonatal CMs (Figures 7A,B). The overexpression of Mettl14 increased the β-catenin level in H_2_O_2_-treated cells compared to that of NC-treated cells, and this effect was significantly counteracted by knockdown of Wnt1 (Figure 7C). The increased cell viability and decline in LDH release caused by Mettl14 in the presence of H_2_O_2_ were attenuated following Wnt1 silencing (Figure 7D). Moreover, knockdown of Wnt1 showed enhanced expression of cleaved caspase-3 in H_2_O_2_-treated cells after the overexpression of Mettl14 (Figure 7E). These data indicate that Mettl14 alleviates cardiomyocyte injury upon oxidative stress *via* activating the Wnt/β-catenin signaling pathway.

## Discussion

Here, we revealed the elevation of m6A levels in parallel with an increase in Mettl14 protein in I/R mice and cardiomyocytes in response to oxidative stress. We found that Mettl14 protected against cardiac I/R injury as indicated by the reduction in apoptosis, cardiac infarct size, and improvement in cardiac dysfunction. Conversely, knockdown of Mettl14 worsened cardiac I/R injury *in vivo* and *in vitro*. Further study showed that Mettl14 was able to lead to an elevation in the Wnt1 protein level by increasing m6A modification of its transcript. These findings suggest that Mettl14 attenuates cardiac I/R injury by activating the Wnt/β-catenin signaling pathway in an m6A-dependent manner (Figure 7G).

m6A modification in RNA has been linked to pathophysiological processes of many diseases due to its specific biological functions. In recent years, many studies have revealed the critical roles of m6A methylation in cardiovascular diseases, which were exemplified by abnormal levels of m6A modification as a result of methyltransferases (writer, Mettl3, and Mettl14) and demethylases (eraser, ALKBH5, and FTO) (Qin et al., 2020). These studies consistently showed that m6A levels were increased in heart failure, fibrosis, and hypertrophy, and cardiomyocyte injury (Dorn et al., 2019; Li et al., 2021; Song et al., 2019). Two different groups have demonstrated that FTO was downregulated in mice failing hearts due to MI, and the overexpression of FTO exerted striking cardioprotection against heart failure by demethylating specific m6A methylated targets (Mathiyalagan et al., 2019; Berulava et al., 2020). Dorn *et al.* (Dorn et al., 2019) demonstrated an increased level of m6A in hypertrophic cardiomyocytes upon pressure overload. Silencing of Mettl3 decreased the m6A methylation of Parp10 mRNA transcripts, thereby upregulating Parp10 protein expression, which in turn led to improvement in cardiomyocyte hypertrophy. In hypoxia/reoxygenation (H/R)-treated cardiomyocytes, m6A levels were increased due to an increase in Mettl3 and a decrease in ALKBH5. The overexpression of ALKBH5 or inhibition of Mettl3 ameliorated H/R-induced cardiomyocytes injury by decreasing m6A modification of TFEB mRNA but increasing the TFEB protein level, a regulator of autophagy (Song et al., 2019). In accordance with these studies, our data also demonstrated that m6A levels were increased in I/R mice and H_2_O_2_-treated neonatal CMs, and this increase was due to the upregulation of Mettl14. This evidence prompted us to determine if Mettl14-mediated m6A modification is implicated in the pathogenesis of I/R injury. Interestingly, in contrast to the evidence that elevation in m6A levels contributes to cardiac injury in many cardiac diseases, our results demonstrated that Mettl14 protected against cardiac I/R injury by employing gain- and loss-of-functional assay. The mechanistic study further indicated that Mettl14 could explain these cardioprotective effects increased m6A modification of Wnt1 mRNA, thereby causing an upregulation of Wnt1 protein and subsequent activation of the Wnt/β-catenin signaling pathway. This implied that m6A-catalyzed enzymes (writers and erasers) affected cardiac pathologies by modifying their specific methylated targets.

The m6A methylated transcripts must be recognized by reader proteins, exerting different biological functions including RNA splicing, stability, and translation. These reader proteins are members of the YT521-B homology (YTH) domain family, consisting of YTH domain family protein 1 (YTHDF1), YTHDF2, and others. As the first m6A readers reported, YTHDF2 recruits CCR4-NOT deadenylase complex after recognizing m6A modified transcripts and eventually transport these RNAs to the processing body to promote RNA degradation (Du et al., 2016). Unlike YTHDF2, YTHDF1 can bind to m6A sites at the region of stop codons and the translation initiation factors and eventually enhance the translation of target transcripts (Wang et al., 2015), which will not result in alteration of RNA stability. Additionally, insulin-like growth factor 2 mRNA–binding proteins (IGF2BPs, including IGF2BP1/2/3) were also identified as m6A reader proteins, promoting mRNA stability and translation in an m6A-dependent manner after binding to m6A sites (Huang et al., 2018). Our results indicated that Wnt1 is a key target for Mettl14-mediated m6A modifications. We found that the overexpression of Mettl14 in cardiomyocytes increased the m6A modification level of Wnt1, and knockdown of Mettl14 reduced the level of Wnt1 m6A modification in cardiomyocytes upon oxidative stress. Notably, Mettl14 failed to alter the expression level of Wnt1 but significantly increased Wnt1 protein levels. A recent study showed YTHDF1 was an amplifier of the Wnt/β-catenin signal at the translation level by promoting the translation of Wnt signal effectors, including TCF7L2/TCF4 (B. Han et al., 2020). According to our results, we speculated that Mettl14 promoted protein translation by increasing Wnt1 m6A modification, which might be mediated by YTHDF1. The underlying mechanism requires additional study to explore.

The activation of the canonical Wnt signaling pathway is indicated by β-catenin stability and nuclear translocation, which interacts with TCF/LEF transcription factors, thereby promoting gene transcription (MacDonald et al., 2009). It was reported that Wnt/β-catenin was activated in fibroblasts, endothelial cells, and progenitor cells within the heart in MI and TAC models (Oerlemans et al., 2010; Akhmetshina et al., 2012; Duan et al., 2012). Wnt signaling exerted different regulatory roles in a different context of pathological processes. The activation of Wnt/β-catenin upon cardiac ischemia enhanced cardiac repair by promoting cardiac fibroblasts to proliferate (Duan et al., 2012). However, the induction of Wnt/β-catenin signaling in cardiac resident fibroblast has been demonstrated to cause cardiac fibrosis in TAC-induced pressure overload. In sharp contrast, activating the Wnt/β-catenin pathway can reduce damage in multiple organs after I/R (Lehwald et al., 2011; Zhu et al., 2018; Yang et al., 2019). Wnt1 caused β-catenin stabilization that functions as a co-activator of HIF-1α signaling, which eventually enhanced hepatocyte survival and protected against hepatic I/R injury (Yang et al., 2019). miR-214 could ameliorate kidney injury by inhibiting apoptosis by activating Wnt/β-catenin signaling (Zhu et al., 2018). Wnt/β-catenin signaling has been reported to be inactive, as indicative of a reduction in Wnt1 and β-catenin protein levels in mouse heart post-I/R (Yang et al., 2019). Silencing miR-148b can increase the survival rate of cardiomyocytes and inhibit cardiomyocyte apoptosis by activating the Wnt/β-catenin signaling pathway, thereby reducing myocardial I/R damage (Yang et al., 2019). In line with this finding, we showed that Wnt/β-catenin signaling was repressed in cardiomyocytes in I/R-treated hearts and by oxidative stress stimuli, and this was reactivated by the overexpression of Mettl14 by inducing Wnt1 protein expression in I/R-treated mice, while Mettl14 knockout exhibited diminishment of Wnt/β-catenin signaling. This supported our conclusion that Mettl14-mediated cardioprotective effects against I/R injury were at least partially due to the activation of Wnt/β-catenin and subsequent apoptosis inhibition.

In conclusion, our study characterizes a novel link between Mettl14-mediated m6A modification and Wnt/β-catenin signaling in the process of I/R-injury. We found that Mettl14 activates the Wnt/β-catenin signaling pathway by upregulating Wnt1 protein expression by methylating Wnt1 mRNA in an m6A-dependent manner, thereby reducing cardiomyocyte injury and cardiac dysfunction upon I/R. Therefore, Mettl14 might serve as a promising therapeutic target for ischemic heart disease.

## Data Availability

'The datasets presented in this study can be found in online repositories. The names of the repository/repositories and accession number(s) can be found below: GEO Curator. with accession GSE186358. (https://www.ncbi.nlm.nih.gov/geo/query/acc.cgi?acc=GSE186358).
