# Supplementary material for: Mettl14 Attenuates Cardiac Ischemia/Reperfusion Injury by Regulating Wnt1/β-Catenin Signaling Pathway
Source: Front Cell Dev Biol. 2021 Dec 16;9:762853. doi: 10.3389/fcell.2021.762853 (PMC8733823; doi:10.3389/fcell.2021.762853)
Supplement: Supplementary file 1 [file DataSheet1.docx]

**Supplementary Figures**

**Supplementary Figure 1**


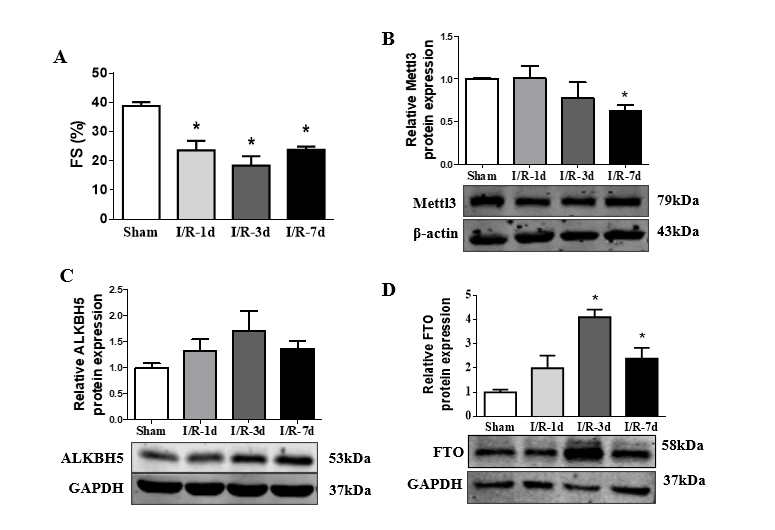


**Supplementary Figure 1. Expression of m6A modification related enzymes at different time points of ischemia and reperfusion(I/R).**

1. Echocardiographic assessment of fractional shortening (FS) in C57BL/6 mice for 1 day, 3 days and 7 days after ischemia and reperfusion or sham operated. Sham n=8, I/R-1d n=6, I/R-3d n=7, I/R-7 n=6. *P＜0.05 versus Sham. P values were determined by unpaired t test. Data are represented as mean ± SEM.
2. Protein level of Mettl3 in I/R-1day, I/R-3day and I/R-7day mice were analyzed by western blotting. Sham n=5, I/R-1d n=5, I/R-3d n=3, I/R-7 n=3. *P＜0.05 versus Sham. P values were determined by unpaired t test. Data are represented as mean ± SEM.
3. Protein level of ALKBH5 in I/R-1day, I/R-3day and I/R-7day mice were analyzed by western blotting. Sham n=5, I/R-1d n=4, I/R-3d n=3, I/R-7 n=4. *P＞0.05 versus Sham. P values were determined by unpaired t test. Data are represented as mean ± SEM.
4. Protein level of FTO in I/R-1day, I/R-3day and I/R-7day mice were analyzed by western blotting. Sham n=4, I/R-1d n=4, I/R-3d n=4, I/R-7 n=5. *P＜0.05 versus Sham. P values were determined by unpaired t test. Data are represented as mean ± SEM.

**Supplementary Figure 2.**


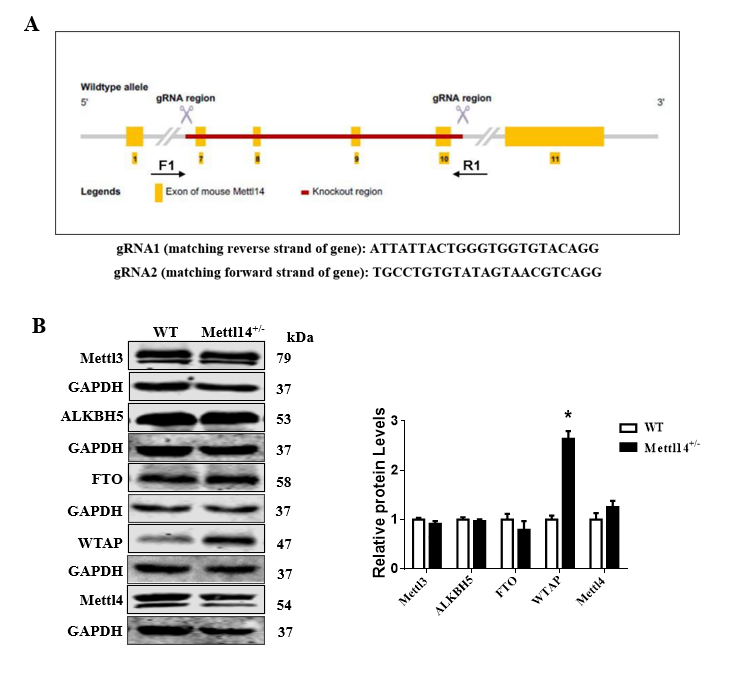


**Supplementary Figure 2. Mettl14 does not affect the expression of m6A-catalyzing complex, except WTAP.**

1. The genotyping strategy of Mettl14+/- mice. Mettl14 heterozygous mice (Mettl14+/-, 8-10 weeks) were generated on the C57/BL6 background by using CRISPR/Cas9-based targeting and homology-directed repair, 7-10 exon was affected. The targeted region of the Mettle14 gene were: gRNA1 (matching the forward strand of gene): ATTATTACTGGGTGGTGTACAGG; gRNA2 (matching the reverse strand of gene): TGCCTGTGTATAGTAACGTCAGG. Mettl14 is not a truncated protein in Mettl14+/- mice.
2. Protein level of Mettl3, ALKBH5, FTO, WTAP, Mettl4 in wild type mice or Mettl14^+/-^ mice were analyzed by western blotting. n=3 mice per group. *P＜0.05 versus WT. P values were determined by unpaired t test. Data are represented as mean ± SEM.

**Supplementary Figure 3.**


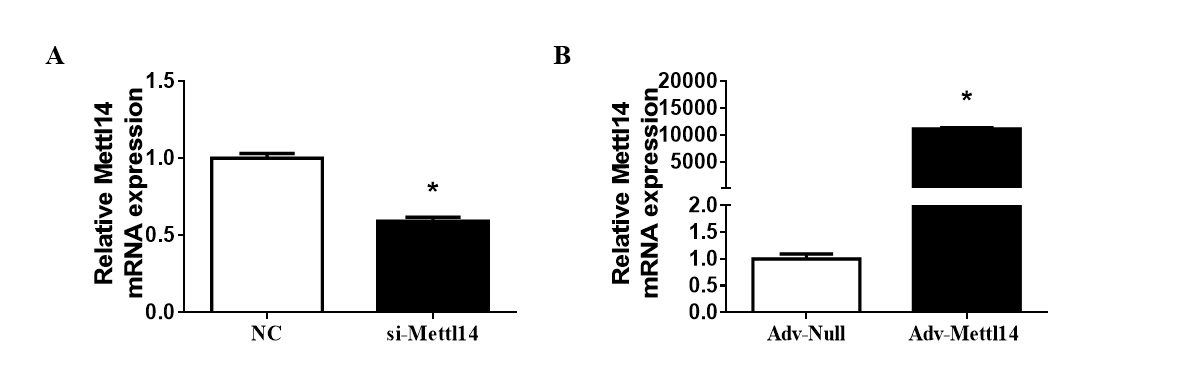


**Supplementary Figure 3. Verification of transfection efficiency: Mettl14 was knocked down by si-RNA (A) and overexpressed by Adv-Mettl14 (B).**

1. Cardiomyocytes were transfected by si-Mettl14. Mettl14 mRNA level in cardiomyocytes was analyzed by qRT-PCR. n=3 mice per group. *P＜0.05 versus NC. P values were determined by unpaired t test. Data are represented as mean ± SEM.
2. Cardiomyocytes was infected with Mettl14 expression vector or empty vector. Mettl14 mRNA level in cardiomyocytes was analyzed by qRT-PCR. n=3 mice per group. *P＜0.05 versus Adv-Null. P values were determined by unpaired t test. Data are represented as mean ± SEM.
